# Pancytopenia With Hypocellular Bone Marrow Revealing Extrahepatic Portal Venous Obstruction and Cavernous Transformation in a Child: A Case Report of a Diagnostic Challenge

**DOI:** 10.1002/ccr3.72948

**Published:** 2026-06-12

**Authors:** Swekchha Adhikari, Aakash Pandit, Melisha Koirala, Biplav Lamichhane, Ram Khadka, Aashish Panta, Shuvra Rimal

**Affiliations:** ^1^ Chitwan Medical College Teaching Hospital Bharatpur Chitwan Nepal; ^2^ National Medical College Teaching Hospital Birgunj Nepal

**Keywords:** case report, cavernous transformation, EHPVO, hypersplenism, literature review, pancytopenia, pediatric age group, portal hypertension

## Abstract

Extrahepatic portal venous obstruction (EHPVO) is a leading cause of prehepatic portal hypertension in children, particularly in developing countries. While upper gastrointestinal bleeding is the most common presentation, EHPVO may rarely manifest predominantly with hematological abnormalities due to hypersplenism, posing a diagnostic challenge and mimicking primary bone marrow disorders. We report a 13‐year‐old female child who presented with pancytopenia, splenomegaly, and easy fatigability, without a history of gastrointestinal bleeding or liver disease. Initial evaluation suggested a primary hematologic disorder, and bone marrow aspiration revealed a hypocellular marrow without malignant infiltration. Extensive workup excluded nutritional, infectious, autoimmune, and malignant causes of pancytopenia. Subsequent abdominal imaging demonstrated non‐visualization of the main portal vein with multiple periportal collateral vessels and hepatopetal flow, consistent with cavernous transformation of the portal vein secondary to chronic extrahepatic portal venous obstruction. Upper gastrointestinal endoscopy revealed grade III esophageal varices, which were managed with endoscopic variceal ligation and initiation of non‐selective beta‐blockers. This case illustrates an uncommon but clinically important presentation of pediatric EHPVO, in which hypersplenism leads to pancytopenia and secondary bone marrow suppression, closely mimicking primary marrow failure. Recognition of this pattern is crucial to avoid misdiagnosis and unnecessary investigations. Imaging, particularly Doppler ultrasonography, plays a pivotal role in establishing the diagnosis. EHPVO should be considered in the differential diagnosis of pancytopenia with splenomegaly in children, even in the absence of overt gastrointestinal bleeding. Early recognition through appropriate imaging can guide timely management and prevent complications of portal hypertension.

## Key Clinical Message

Pancytopenia in children may be the initial manifestation of extrahepatic portal vein obstruction due to hypersplenism. Careful clinical evaluation combined with Doppler imaging and endoscopic assessment is essential to distinguish secondary marrow suppression from primary hematologic disorders and to prevent complications of portal hypertension.

## 1 Introduction

Extrahepatic portal venous obstruction (EHPVO) is a major cause of prehepatic portal hypertension in children, particularly in developing countries, and often occurs in the absence of intrinsic liver disease. Pediatric EHPVO accounts for the majority of non‐cirrhotic portal hypertension cases and is characterized by obstruction of the extrahepatic portal vein, leading to the development of collateral veins around the site of obstruction, termed cavernous transformation of the portal vein (CTPV) [1, 2].

The pathophysiology of EHPVO involves thrombotic occlusion of the portal vein outside the liver, which diverts portal blood flow through a network of periportal and collateral vessels in an attempt to maintain hepatopetal flow. These tortuous collateral channels form a cavernoma, detectable on Doppler ultrasonography or CT/MR angiography [2, 3].

Clinically, EHPVO in children presents with a broad spectrum of symptoms related to portal hypertension, including splenomegaly, variceal bleeding, and hypersplenism. Splenomegaly and its hematological sequelae, particularly thrombocytopenia, anemia, and leukopenia, may dominate the clinical picture, sometimes preceding upper gastrointestinal hemorrhage [1, 4].

Hypersplenism in portal hypertension is a well‐recognized phenomenon whereby an enlarged spleen sequesters and destroys circulating blood elements, resulting in pancytopenia. This hematologic pattern may prompt an initial suspicion of primary bone marrow pathology, often necessitating extensive hematologic evaluation, including bone marrow aspiration and biopsy [5].

While upper gastrointestinal bleeding from esophageal and gastric varices is the most common acute presentation of pediatric EHPVO, non‐bleeding manifestations such as anemia, hypersplenism with cytopenias, growth retardation, and portal biliopathy are increasingly recognized in children [6].

The diagnosis of EHPVO is generally established by imaging modalities such as Doppler ultrasonography, which demonstrates absence or occlusion of the main portal vein with formation of cavernous collaterals, supplemented by CT or MR angiography to delineate the portal venous anatomy [7].

Management of EHPVO focuses on controlling complications of portal hypertension. Endoscopic therapy and non‐selective β‐blockers are used for variceal bleeding prophylaxis, while surgical procedures such as the Rex shunt can restore portal venous flow, reduce portal pressure, and ameliorate hypersplenism [7, 8].

Despite being one of the more common etiologies of pediatric portal hypertension in resource‐limited settings, EHPVO remains under‐recognized when presenting primarily with hematological abnormalities such as pancytopenia and hypocellular bone marrow, which can mimic primary hematologic disorders [9].

In this report, we describe a 13‐year‐old female child who presented with pancytopenia and marked splenomegaly, initially raising concern for a primary bone marrow disorder. Subsequent evaluation revealed a hypocellular bone marrow in the absence of malignant infiltration and imaging evidence of chronic portal and splenic vein thrombosis with cavernous transformation of the portal vein, consistent with extrahepatic portal venous obstruction. This case highlights an uncommon but important presentation of pediatric EHPVO, in which hypersplenism and portal hypertension masqueraded as bone marrow failure, leading to extensive hematologic evaluation before the underlying diagnosis was established.

## 2 Case History/Examination

A 13‐year‐old female child presented with a one‐month history of right upper quadrant abdominal pain, described as dull‐aching, intermittent, non‐radiating, and of mild to moderate intensity, without identifiable aggravating or relieving factors. She also reported easy fatigability but denied fever, vomiting, jaundice, hematemesis, melena, altered bowel habits, or weight loss. There was no history of recurrent infections, night sweats, bone pain, rashes, or joint pain. She had no history of umbilical catheterization or neonatal intensive care admission.

The past medical history was notable for multiple outpatient visits over the preceding month, during which splenomegaly with pancytopenia had been documented. Hospital admission for further evaluation was initially advised but declined due to financial constraints. Birth history revealed a single‐term normal vaginal delivery at a peripheral health post, with a birth weight of 1900 g. Immunizations were complete according to the national EPI schedule in Nepal. Family history was significant for consanguinity (parents were first cousins) and pulmonary tuberculosis in a grandmother, while no hematologic disorders were reported.

On general examination, the patient appeared well‐built and alert, with pallor. No jaundice was noted. A single palpable left level III cervical lymph node measuring 2 cm was identified; it was mobile, non‐tender, and without overlying skin changes. Given the absence of fever, night sweats, hepatosplenomegaly‐related lymphatic congestion, and systemic symptoms, this node was considered most likely reactive in etiology. No dedicated neck imaging or nodal biopsy was performed, as the clinical picture was not suggestive of lymphoma or tuberculosis‐related lymphadenopathy, and the node remained stable during the admission period. There were no stigmata of chronic liver disease. Anthropometric measurements were weight 43 kg, height 156 cm, and BMI 18.3 kg/m^2^.

Vital signs were within normal limits, with temperature 97°F–98.6°F, pulse 95–96 bpm, respiratory rate 18–22/min, blood pressure 100/60 mmHg, and SpO_2_ 100% on room air.

Systemic examination revealed a soft, non‐tender abdomen with a palpable spleen 5–6 cm below the left costal margin; the liver was not palpable and there was no ascites. Cardiovascular and respiratory examinations were normal. Neurologic evaluation demonstrated normal tone, reflexes, and no focal deficits. Musculoskeletal examination showed a mild deformity of the right elbow joint without bony tenderness. The skin was unremarkable, with no rashes or signs of portal hypertension such as spider angiomata.

## 3 Methods (Investigation and Treatment)

A comprehensive evaluation was undertaken to determine the cause of pancytopenia and splenomegaly. Serial complete blood counts revealed persistent pancytopenia with WBC ranging from 2400 to 2830/μL, hemoglobin 9.6–10.8 g/dL, and platelets 53,000–105,000/μL. Peripheral blood smear showed normocytic normochromic red blood cells with occasional elliptocytes, teardrop cells, and target cells, alongside decreased leukocytes and platelets but no atypical cells. Reticulocyte counts were low‐normal. Hemolytic, enzymatic, and nutritional causes were systematically excluded with negative Coombs test, normal G6PD activity, normal vitamin B12 levels, and hemoglobin electrophoresis, while infectious causes including HIV, malaria, and Leishmania donovani were ruled out. Liver function parameters including transaminases, bilirubin, albumin, and coagulation profile were within normal limits, excluding intrinsic hepatocellular disease and supporting a diagnosis of prehepatic portal hypertension.

Bone marrow aspiration was performed from the posterior superior iliac spine to evaluate primary marrow pathology. The aspirate demonstrated hypocellular marrow with suppressed erythropoiesis and myelopoiesis, 10% megaloblastoid changes, and decreased megakaryocytes, along with increased lymphopoiesis and < 1% plasma cells. There was no evidence of malignant infiltration or parasitic involvement, indicating secondary marrow suppression. Notably, this hypocellular pattern is atypical, as hypersplenism classically elicits a compensatory hypercellular marrow response; the hypocellularity observed here likely represents an unusual manifestation requiring further mechanistic explanation.

Given the clinical suspicion of a prothrombotic state, thrombophilia screening including Protein C, Protein S, and Factor V Leiden testing was indicated; however, this was not feasible due to significant financial constraints and limited laboratory access in our resource‐limited setting. The absence of this workup introduces uncertainty regarding the underlying etiologic mechanism of portal vein thrombosis. Identification of an inherited thrombophilic state would have important implications for long‐term anticoagulation decisions, recurrence risk counseling, and screening of first‐degree relatives, particularly relevant given the documented parental consanguinity in this case. This gap underscores the need for accessible thrombophilia testing in pediatric portal hypertension workup in low‐resource settings.

Imaging studies were critical in establishing the underlying etiology. Abdominal ultrasound and CT scan revealed marked splenomegaly (17.7–18.5 cm) with non‐visualization of the main portal vein, which was replaced by cavernous transformation. The splenic vein and superior mesenteric vein (SMV) were noted to be patent but significantly dilated (> 10 mm), indicative of high back‐pressure and congestive splenomegaly. Multiple retroperitoneal and mesenteric lymph nodes were noted, the largest measuring 15 × 10 mm. Doppler ultrasonography confirmed hepatopetal flow through the collateral vessels. Hepatic artery and vein flow patterns were normal, and there was no focal hepatic lesion.

Endoscopic evaluation revealed grade III esophageal varices with red color signs, for which five bands were deployed, along with GOV‐1 varices without red color signs. Management focused on controlling portal hypertension and preventing variceal bleeding, with initiation of non‐selective beta‐blockers for secondary prophylaxis. Supportive care included monitoring of blood counts, nutritional supplementation, and counseling on bleeding precautions. Future surgical intervention, such as a Rex shunt, was considered in case of progressive hypersplenism, recurrent variceal bleeding, or worsening cytopenias. Close outpatient follow‐up with repeat imaging, endoscopic surveillance, and hematologic evaluation was planned to monitor disease progression and response to treatment.

Management focused on the prevention of variceal bleeding. Following endoscopic band ligation, the patient was initiated on propranolol for secondary prophylaxis of variceal bleeding. Propranolol was started at 1 mg/kg/day in two divided doses and titrated to achieve a resting heart rate reduction of 25% from baseline, or a target heart rate of approximately 55–60 bpm, while maintaining hemodynamic stability. The patient was monitored for adverse effects including bradycardia, hypotension, bronchospasm, and hypoglycemia, the last being of particular concern in pediatric patients. While evidence for non‐selective beta‐blockers in pediatric EHPVO is largely extrapolated from adult trials, their use in children with high‐risk varices is accepted practice with close clinical monitoring [10]. No blood transfusion was required during admission, as hemoglobin remained above 9.6 g/dL throughout; transfusion thresholds were set at hemoglobin below 7 g/dL or symptomatic anemia, in keeping with standard supportive care guidelines.

## 4 Conclusion and Results (Outcome and Follow‐Up)

Following endoscopic variceal ligation and initiation of non‐selective beta‐blockers, the patient remained clinically stable with no new episodes of gastrointestinal bleeding. Pancytopenia showed relative stability, and no additional complications were reported during the immediate post‐procedure period. She was discharged with instructions for close outpatient monitoring, including endoscopic surveillance, repeat imaging of the portal and splenic vasculature, and regular hematologic evaluation. However, the patient did not return for further follow‐up so long‐term outcomes could not be assessed.

## 5 Differential Diagnosis

The presentation of pancytopenia with splenomegaly in a child is multifactorial, requiring consideration of hematologic, infectious, metabolic, and vascular etiologies. In this patient, the following differential diagnoses were critically considered:

### 5.1 Primary Bone Marrow Disorders

- Aplastic anemia: Typically presents with pancytopenia and hypocellular marrow. However, in this patient, bone marrow was hypocellular but showed evidence of secondary suppression, and there was a clear vascular etiology (portal vein obstruction) causing splenic sequestration. No congenital or autoimmune features suggestive of primary aplastic anemia were present.
- Acute leukemia or myelodysplastic syndrome: These can present with pancytopenia and splenomegaly. Peripheral smear and bone marrow showed no blasts or dysplastic lineages beyond mild megaloblastoid changes, arguing against a primary malignant process.

### 5.2 Hypersplenism Secondary to Portal Hypertension

- Extrahepatic portal vein obstruction (EHPVO): Fits the clinical and imaging findings perfectly, including non‐visualization of the main portal vein, cavernous transformation, multiple collateral vessels, and splenomegaly. The bone marrow showed secondary suppression consistent with peripheral sequestration, making this the most likely etiology.

### 5.3 Chronic Infections

- Visceral leishmaniasis: Can cause pancytopenia and splenomegaly, particularly in endemic regions. However, Leishmania serology was negative, and there was no relevant travel or exposure history.
- Malaria: Can occasionally lead to splenomegaly and cytopenias, but blood smears were repeatedly negative, and there were no febrile episodes.
- Chronic viral hepatitis or HIV: Laboratory testing excluded these as causes.

### 5.4 Congenital or Metabolic Disorders

- Gaucher disease: Can present with splenomegaly and cytopenias. However, the absence of hepatomegaly, bone pain, or characteristic storage cells on bone marrow makes this unlikely.
- Other storage disorders (Niemann‐Pick, etc.): Also unlikely due to normal neurological and developmental examination and absence of typical marrow or imaging findings.

### 5.5 Autoimmune or Hematologic Disorders

- Autoimmune cytopenias: Coombs tests were negative, and there was no history of hemolysis, jaundice, or autoimmune disease, making immune‐mediated cytopenias improbable.

### 5.6 Vascular or Structural Liver Disease

- Budd‐Chiari syndrome: Usually presents with hepatomegaly, ascites, and liver function abnormalities. In this patient, liver size and function were normal, and imaging confirmed portal vein obstruction without hepatic vein involvement.
- Congenital hepatic fibrosis or cirrhosis: Imaging and laboratory evaluation did not support chronic liver disease, and there were no stigmata of chronic liver pathology.

## 6 Discussion

The diagnostic trajectory in this case followed a pattern likely to be encountered by clinicians in resource‐limited settings: an initial presentation with pancytopenia and splenomegaly prompted extensive hematologic investigation, including peripheral smear analysis and bone marrow aspiration, with primary bone marrow failure as the leading concern. The absence of malignant cells, the non‐diagnostic marrow pattern, and the failure to identify infectious or autoimmune causes prompted clinical reconsideration. Only at this stage was abdominal imaging prioritized, which revealed cavernous transformation of the portal vein and established the diagnosis of EHPVO. This sequence initial hematologic focus, systematic exclusion, and delayed vascular imaging reflects a common but avoidable diagnostic pitfall, and highlights the importance of incorporating Doppler ultrasonography early in the evaluation of any child presenting with unexplained pancytopenia and splenomegaly. We recommend that Doppler ultrasonography of the portal venous system be performed early ideally as part of the initial assessment in any child presenting with otherwise unexplained pancytopenia accompanied by splenomegaly. This non‐invasive, widely available modality can rapidly identify portal vein occlusion and cavernous transformation, potentially sparing the child from invasive bone marrow procedures and significantly shortening the diagnostic interval. Extrahepatic portal vein obstruction (EHPVO) is a well‐recognized cause of prehepatic portal hypertension in children, frequently presenting with splenomegaly, esophageal varices, and hypersplenism. EHPVO results from thrombotic or obstructive occlusion of the extrahepatic portal vein, leading to the development of a network of collateral vessels termed cavernous transformation (portal cavernoma). These collateral veins bypass the obstructed segment of the portal vein to maintain hepatopetal blood flow, but in doing so create persistent portal hypertension and its complications [8, 11].

In pediatric cohorts, EHPVO is the most common cause of non‐cirrhotic portal hypertension and may occur idiopathically or in association with risk factors such as umbilical vein catheterization, neonatal sepsis, dehydration, and inherited thrombophilic states [1, 12]. In one large pediatric series, splenomegaly and hypersplenism were documented in more than 60% of children with EHPVO, underlining the frequency with which hematologic abnormalities can be the presenting feature [1].

Hypersplenism in the context of portal hypertension develops as a consequence of increased portal venous pressure, which leads to congestion and enlargement of the spleen. The enlarged spleen sequesters and destroys circulating blood elements, resulting in peripheral cytopenias involving white blood cells, red blood cells, and platelets. Although hypersplenism is classically associated with thrombocytopenia, anemia and leukopenia can also occur, producing a pancytopenic picture [13]. While bone marrow aspiration in hypersplenism classically demonstrates compensatory hypercellularity due to peripheral destruction, marrow hypocellularity as observed in our patient is an atypical and uncommonly reported finding in this context. Lv et al. [5] noted that chronic portal hypertension may alter hematopoietic output through mechanisms beyond simple sequestration, supporting the possibility that prolonged portal hypertension can paradoxically suppress marrow activity. To our knowledge, few pediatric case reports have explicitly documented hypocellular marrow as a presenting feature of EHPVO‐related hypersplenism. Salloum et al. [9] described an atypical portal vein thrombosis presentation in a toddler, and Gökçay et al. [1] emphasized the varied hematologic presentations of EHPVO in children, but neither specifically addressed hypocellular marrow morphology. This case therefore contributes a novel and cautionary observation to the literature: that secondary marrow hypocellularity, rather than the expected hyperplasia, may occur in the context of chronic portal hypertension and profound hypersplenism, representing an underappreciated diagnostic mimicker of primary bone marrow failure.

Esophageal and gastric varices are common complications of EHPVO because persistent portal hypertension leads to development of portosystemic collaterals at sites of low resistance, particularly the gastroesophageal junction. Upper gastrointestinal bleeding from varices is frequently the first recognized clinical complication, although many children with EHPVO present with splenomegaly and cytopenias without overt bleeding [1, 7]. Doppler ultrasonography plays a central role in diagnosis by revealing non‐visualization of the portal vein and cavernous transformation, with hepatopetal flow through collaterals, as seen in this case [7].

Management of pediatric EHPVO is primarily directed at the sequelae of portal hypertension. Endoscopic variceal ligation (EVL) and/or sclerotherapy effectively control and prevent esophageal variceal bleeding and are considered standard therapeutic modalities when varices with high‐risk stigmata are present [10].

Non‐selective beta‐blockers are frequently used for secondary prophylaxis of variceal bleeding, though evidence in children is extrapolated from adult data due to limited pediatric trials [10].

In cases of recurrent bleeding, refractory hypersplenism, or growth failure, surgical interventions such as the Rex shunt or other portal decompressive procedures may be considered to restore physiological portal flow and reduce portal pressure. A recent single‐center pediatric series by Chen et al. [8] demonstrated favorable outcomes following Rex shunt in children with portal vein cavernous transformation, with resolution of hypersplenism and improvement in cytopenias in the majority of cases, further supporting early surgical referral when clinically indicated.

This case illustrates an unusual and diagnostically challenging phenomenon: the coexistence of hypersplenism‐driven peripheral destruction with a hypocellular rather than hypercellular bone marrow. While compensatory marrow hyperplasia is the expected response to peripheral cytopenias, the hypocellularity observed here may be hypothetically attributed to chronic elevation of pro‐inflammatory cytokines such as TNF‐alpha, or progressive exhaustion of the hematopoietic niche under sustained peripheral demand mechanisms proposed in the literature on chronic liver disease and portal hypertension [5, 13]. These remain hypotheses in this case, given the absence of confirmatory cytokine or marrow biopsy data, and warrant further investigation in future studies.

The identification of cavernous transformation of the portal vein (CTPV) via Doppler ultrasonography provides highly suggestive and diagnostically supportive evidence that should prompt a shift in clinical focus from primary hematologic evaluation toward portal hypertension management [7, 11]. While endoscopic ligation and pharmacological interventions are standard for immediate variceal prophylaxis [10], the Meso‐Rex bypass (Rex shunt) remains the most physiological long‐term solution by restoring hepatopetal flow and addressing the root cause of congestive splenomegaly [8].

However, as demonstrated in this case, the challenges of resource‐limited settings including financial barriers and loss to follow‐up often impede access to such definitive surgical interventions [1, 6].

## 7 Conclusion

This case highlights extrahepatic portal vein obstruction as an important and often overlooked cause of pancytopenia in children, mediated through hypersplenism. In the absence of intrinsic liver disease, early recognition of portal hypertension using Doppler imaging and endoscopy is crucial to prevent life‐threatening complications such as variceal bleeding. The loss to follow‐up underscores the socioeconomic challenges inherent in managing chronic pediatric conditions in developing regions. This report emphasizes the importance of a systematic diagnostic approach to pancytopenia in pediatric patients, particularly in resource‐limited settings where delayed diagnosis remains common.

## Author Contributions

**Swekchha Adhikari:** conceptualization, supervision, validation, writing – original draft, writing – review and editing. **Aakash Pandit:** conceptualization, formal analysis, writing – original draft, writing – review and editing. **Melisha Koirala:** supervision, validation, writing – original draft, writing – review and editing. **Aashish Panta:** data curation, writing – original draft, writing – review and editing. **Biplav Lamichhane:** data curation, writing – original draft, writing – review and editing. **Ram Khadka:** data curation, writing – original draft, writing – review and editing. **Shuvra Rimal:** data curation, writing – original draft, writing – review and editing.

## Funding

The authors have nothing to report.

## Consent

Written informed consent was obtained from the patient's parents/legal guardian for publication and any accompanying images. A copy of the written consent is available for review by the Editor‐in‐Chief of this journal on request.

## Conflicts of Interest

The authors declare no conflicts of interest.

## Data Availability

The data that support the findings of this study are available from the corresponding author upon reasonable request.
